# Pancreatic panniculitis associated with pancreatic carcinoma: A case report

**DOI:** 10.1097/MD.0000000000004374

**Published:** 2016-08-07

**Authors:** Guannan Zhang, Zhe Cao, Gang Yang, Wenming Wu, Taiping Zhang, Yupei Zhao

**Affiliations:** Department of General Surgery, Peking Union Medical College Hospital, Chinese Academy of Medical Sciences and Peking Union Medical College, Beijing, China.

**Keywords:** pancreatic mucinous adenocarcinoma, pancreatic panniculitis

## Abstract

**Introduction:**

Pancreatic panniculitis is a very rare complication of pancreatic cancer, most often accompanying rare acinar cell carcinoma. We herein report a case of pancreatic panniculitis that was associated with pancreatic mucinous adenocarcinoma.

**Patient information:**

A 57-year-old male was referred to our hospital for weight loss. A physical examination revealed subcutaneous nodules on his lower extremities. The blood test showed abnormal increases in amylase, lipase, and carbohydrate antigen 19–9 levels. A computed tomography scan detected a hypodense 2 × 1.5 cm solid mass with an unclear margin in the head of the pancreas. The biopsy of subcutaneous nodules on the lower extremities was conducted and revealed lobular panniculitis. Pancreatic cancer and pancreatic panniculitis were strongly suspected. After the administration of octreotide acetate and the Whipple procedure, the serous amylase and lipase levels returned to normal, and the pancreatic panniculitis had almost resolved by 4 weeks later.

**Conclusion:**

Pancreatic panniculitis is a rare complication of pancreatic cancer. However, in the presence of a pancreatic mass, as in this case, clinicians should be aware that panniculitis may be the sentinel of pancreatic carcinoma.

## 1 Introduction

Pancreatic panniculitis (PP) is the rare necrosis of subcutaneous fat and occurs in ∼0.3% to 1% of all patients with pancreatic disease.^[1]^ PP has been reported in acute and chronic pancreatitis^[2]^ and pancreatic neoplasms (acinar cell carcinoma in 80% of cases).^[3]^ We herein report a rare case of PP that was associated with pancreatic mucinous adenocarcinoma.

## 2 Case report

A 57-year-old male who complained of multiple subcutaneous nodules on his lower legs for ∼4 months without any other history presented to the hospital due to weight loss that began ∼2 months ago.

**Figure 1:**
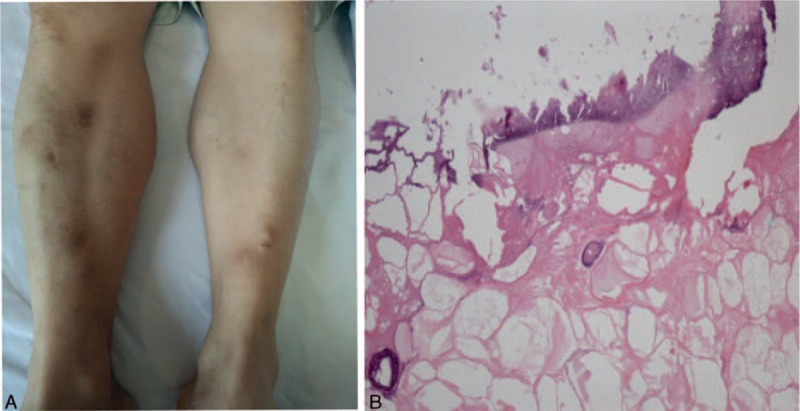
Subcutaneous nodule on the lower extremities. (A) Tender erythematous subcutaneous nodules on the lower extremities; (B) necrosis with nuclear debris and “ghost” cells’ characterized by anucleated adipocytes with partially digested shadowy cell membranes.

A physical examination upon admission revealed multiple edematous erythematous, tender, ill-defined, subcutaneous nodules ∼1.5 cm in diameter with heat and fluctuation on the lower extremities but without swelling or pain (Fig. 1A). No knee or ankle joint pain or abdominal symptoms were detected.

The blood test revealed increases in amylase (AMY) (2161U/L; reference range 25–115U/L), lipase (LIP) (27575U/L; reference range 73–393U/L), carbohydrate antigen 19–9 (CA19–9) (69.8U/mL; reference range 0–34.0 U/mL), gamma-glutamyl transpeptidase (GGT) (463U/L; reference range 10–60U/L), alkaline phosphatase (ALP) (194U/L; reference range 45–125U/L), aspartate transaminase (AST) (71U/L; reference range 15–40U/L), alanine aminotransferase (ALT) (120U/L; reference range 9–50U/L), c-reactive protein (CRP) (16.90 mg/L; reference range 0–3 mg/L) and eosinophil percentage (EOS%) (6.4%; reference range 0.5–5.0%). Albumin (ALB) (162 mg/L; reference range 200–400 mg/L), apolipoprotein-A1 (ApoA1) (0.94 g/L; reference range 1.05–1.75 g/L), and apolipoprotein-B (Apo-B) (0.57 g/L; reference range 0.6–1.4 g/L) levels were slightly decreased. The white blood cell count and IgG and IgG4 levels were normal.

**Figure 2:**
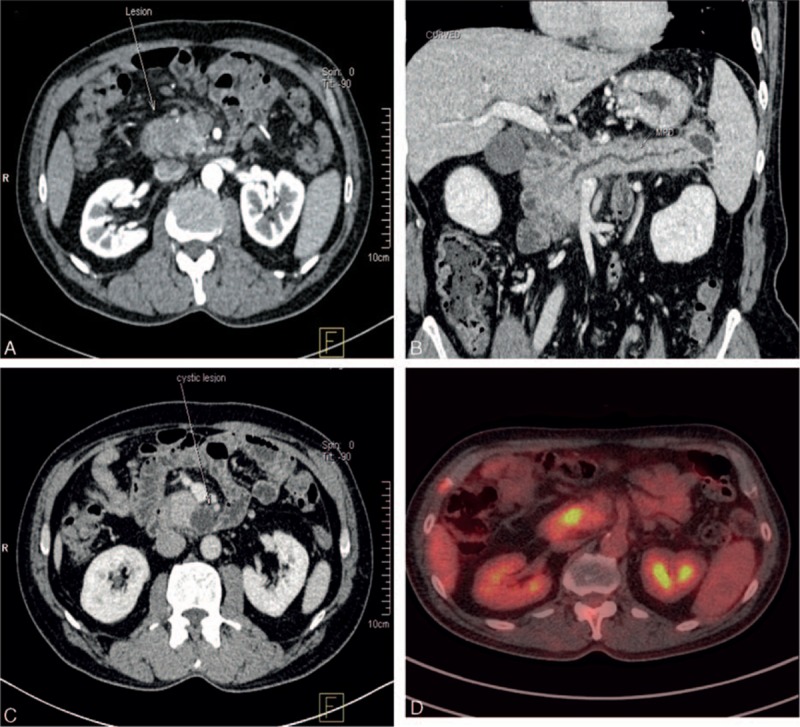
Imaging findings from the pancreatic tumor. (A) Computed tomography detected a 2 × 1.5 cm hypodense solid mass with an unclear margin in the head of the pancreas; (B) expanded primary pancreatic duct and the cystic low density in the tail of pancreas; (C) cystic low density in the uncinate process of the pancreas; (D) PET-CT revealed a malignant mass in the pancreatic head.

A computed tomography (CT) scan detected a hypodense 2 × 1.5 cm solid mass with an unclear margin in the head of the pancreas with homogenous lower enhancement compared to the surrounding pancreatic parenchyma by intravenous contrast in the arterial phase (Fig. 2A). In addition, we observed an expanded primary pancreatic duct and inter- and extra-bile ducts in addition to cholecyst and multiple cystic lesions in the swollen pancreas with rough edges (Fig. 2B and C). Positron emission tomography-computed tomography revealed a malignant mass in the pancreatic head (Fig. 2D). We conducted a biopsy of the subcutaneous nodules on the lower extremities. The pathology results indicated lobular panniculitis with foci of necrosis and “ghost” cells characterized by anucleated adipocytes with partially digested shadowy cell membranes (Fig. 1B). Pancreatic cancer and PP were strongly suspected.

**Figure 3:**
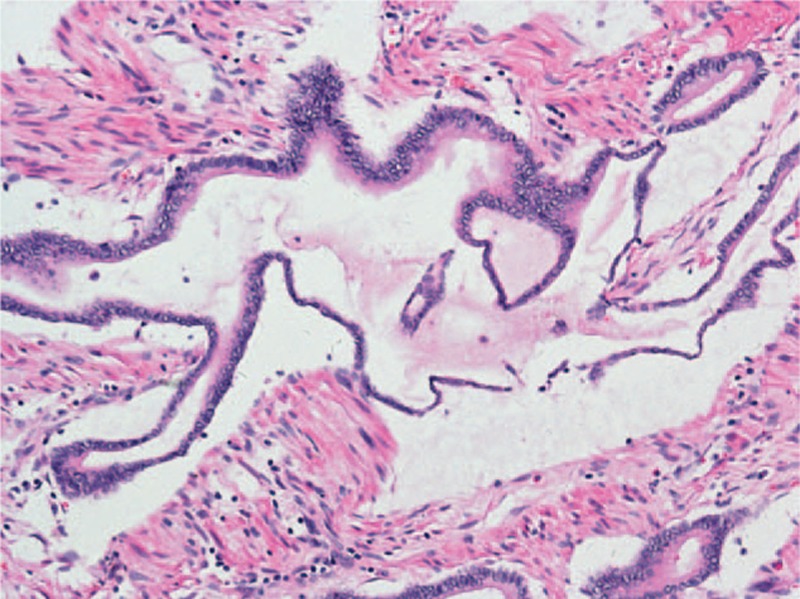
Pathological findings (hematoxylin/eosin staining): pancreatic mucinous adenocarcinoma.

Because of the high levels of AMY and LIP, which increased to 4129U/L and 58412U/L after admission, sandostain (octreotide acetate injection) was administered after obtaining informed consent. The serous AMY and LIP levels decreased to 649U/L and 6170U/L, respectively, 7 days later. Additionally, the size and amount of erythematous subcutaneous nodules on the lower legs decreased. After exhaustive explanation of the condition, the patient underwent the Whipple procedure. A biopsy of the resected tumor revealed mucinous adenocarcinoma (Fig. 3). The serous AMY and LIP levels returned to normal, and the PP had almost resolved 4 weeks later.

## 3 Discussion

PP is the rare necrosis of subcutaneous fat and occurs in ∼0.3% to 1% of all patients with pancreatic disease.^[1]^ PP has been reported in acute and chronic pancreatitis^[2]^ and pancreatic neoplasms (acinar cell carcinoma in 80% of the cases).^[3]^ To the best of our knowledge, this is the first report describing subcutaneous panniculitis associated with pancreatic mucinous adenocarcinoma.

Clinically, panniculitis presents as erythematous, ill-defined, red-brown nodules that are usually located on the lower extremities but can also appear on the arms, trunk, and breasts.^[4]^ Distinctive laboratory values include eosinophilia and raised serum lipase levels, which are related to the advance of PP.

The pathogenesis of PP is not well understood. Exocrine acinar cancer cells produce lipase and other digestive enzymes and may release the hydrolase into circulation. However, ductal adenocarcinoma of the pancreas, such as in this case, may not produce sufficiently high levels of circulating hydrolase to induce subcutaneous fat necrosis. One possible explanation is that pancreatic enzymes may not be the only etiological factor; an immunological process (such as alpha–antitrypsin deficiency) can initiate subcutaneous necrosis.^[5]^

The treatment of PP should be directed to the underlying pancreatic diseases. No other therapeutic agents have been shown to help eliminate the skin eruptions.^[4]^ In this case, PP regressed 2 weeks after the Whipple procedure.

In conclusion, clinicians should be aware that panniculitis may be the sentinel of pancreatic carcinoma and may precede common manifestations.^[6]^ Only a minority of subcutaneous panniculitis cases are associated with pancreatic disease. However, in the presence of a pancreatic mass, as in our case, the diagnosis of carcinoma should strongly be considered.

## References

[1] Bogart MM Milliken MC Patterson JW Pancreatic panniculitis associated with acinic cell adenocarcinoma: a case report and review of the literature. *Cutis* 2007; 80:289–294. 18038690

[2] Jacobson-Dunlop E Takiguchi R White CR Fatal pancreatitis presenting as pancreatic panniculitis without enzyme elevation. *J Cutan Pathol* 2011; 38:455–457. 21521354 10.1111/j.1600-0560.2011.01725_1.x

[3] Moro M Moletta L Blandamura S Acinar cell carcinoma of the pancreas associated with subcutaneous panniculitis. *JOP* 2011; 12:292–296. 21546712

[4] García-Romero D Vanaclocha F Pancreatic panniculitis. *Dermatol Clin* 2008; 26:465–470.vi. 18793978 10.1016/j.det.2008.05.009

[5] Lyon MJ Metabolic panniculitis: alpha-1 antitrypsin deficiency panniculitis and pancreatic panniculitis. *Dermatol Ther* 2010; 23:368–374. 20666824 10.1111/j.1529-8019.2010.01337.x

[6] Marsh Rde W Hagler KT Carag HR Pancreatic panniculitis. *Eur J Surg Oncol* 2005; 31:1213–1215. 16099617 10.1016/j.ejso.2005.06.007

